# Editorial: Genome editing for agricultural sustainability: developments in tools, potential applications, and regulatory policy

**DOI:** 10.3389/fgeed.2023.1324921

**Published:** 2023-10-31

**Authors:** Felicity J. Keiper, Thorben Sprink, Ian Douglas Godwin

**Affiliations:** ^1^ BASF Australia Ltd., Melbourne, VIC, Australia; ^2^ Federal Research Centre for Cultivated Plants, Institute for Biosafety in Plant Biotechnology, Julius Kühn-Institute, Quedlinburg, Germany; ^3^ School of Agriculture and Food Sciences, The University of Queensland, Brisbane, QLD, Australia

**Keywords:** genome editing, agricultural sustainability, plant breeding, crop improvement, regulatory policy

In this Research Topic, nine articles were published, contributing to the expanding corpus recognising the need to adopt innovations in plant breeding to enhance the sustainability of agricultural production and to address broader global issues. In regard to crop improvement, the articles exemplify the application of genome editing in the development of new traits for preventing significant yield losses (Hoffie et al.), examine its application for *de novo* domestication of wild crop relatives (Abdullah et al.), and review the scope and types of genetic changes that are possible with genome editing in comparison to that possible with conventional breeding tools (Martinez-Fortún et al.). The potential contributions to agricultural sustainability are examined in national (Matsuo and Tachikawa), regional (Tripathi et al., Hingsamer et al.) and international (Smyth; Spök et al.) contexts, and the importance of capacity building and an enabling regulatory environment recognised (Smyth; Keiper and Atanassova; Tripathi et al.).

It is evident in these articles that the accessibility of genome editing tools offers great potential for delivering improved varieties, with applications in a diversified range of crops extending beyond staple crops to others that also have an important role in food security. Improved nutrition and food security are elements of the United Nations Sustainable Development Goals (SDGs), which are the focus of Smyth. In this work, developments in applications impacting crop yield and nutrition, and improved sustainability in crop and meat production, are reviewed in the context of their potential contributions to the first three SDGs: ending poverty (#1); zero hunger (#2), which encompasses improved nutrition and food security, and promotion of agricultural sustainability and productivity; and good health and wellbeing (#3). The implications of the regulatory environment for realising potential benefits are also discussed.

Two articles in this Research Topic examine aspects of these global goals in different regional contexts. The review by Tripathi et al. emphasises the need to realise the full potential of all tools available for crop improvement if sustainable intensification of agriculture, along with food security and improved nutrition, are to be achieved in Africa. This work reviews recent developments in genome editing applications for African staple and orphan crops, as well as developments in the applicable regulatory landscape. Hingsamer et al. present a case study on root chicory, which provides an important source of inulin. Drawing on research developing root chicory as a multipurpose crop in the European Union, this work undertakes a socio-economic and environmental impact assessment of the value chain. The findings indicate the potential for new traits developed using genome editing for boosting economic activity and regional agricultural competitiveness, as well as improving process (and energy) efficiencies.

The potential application of genome editing to *de novo* domestication is examined by Abdullah et al. This work reviews recent advances in genome editing in rice, and examples of its application to rapidly introduce beneficial traits in wild plant relatives. While beneficial traits have successfully been transferred from wild rice using conventional tools, limitations of existing approaches could be overcome by editing wild rice homologs of domestication-related genes. The biotic and abiotic stress tolerance traits in Australian wild rices that could contribute to yield improvements in domesticated rice breeding programs are also highlighted.

One of the drivers for optimism around the opportunities and potential benefits of genome editing is an expectation for a more favourable regulatory environment—compared to that for genetically modified (GM) crops—that should facilitate greater technology adoption. This is evident in many of the articles in this Research Topic, with four specifically examining regulatory policy and societal dynamics (Keiper and Atanassova; Matsuo and Tachikawa; Martinez-Fortún et al.; Spök et al.).

The review by Martinez-Fortún et al. analyses spontaneous and induced genetic variation that is generated and selected in conventional plant breeding as a baseline for comparing the genetic changes possible with genome editing. Based on this, they contend that the outcomes of genome editing, specifically products that do not contain transgenes, could have been generated using conventional breeding tools, and regulating them in the same manner as GM products would not be proportionate or appropriate. An example of the regulatory approach taken in a jurisdiction based on this premise is reviewed by Matsuo and Tachikawa. In Japan, three genome edited food products have completed the requisite notification process for commercial use: one plant (GABA-enriched tomato), and two fish. This work examines the factors contributing to societal acceptance of these products, as contrasted with the historical controversy accompanying GM crops. The authors note shifts in societal values, including greater awareness of sustainability, but also that these are niche products and that food crops for mass production and trade may face a greater test.

The review by Spök et al., takes a deep dive into genome editing regulatory policy developments and their drivers in “adopter” (of commercial GM crop cultivation) and “non-adopter” jurisdictions throughout the world. They report 18 jurisdictions have either exempted certain genome edited organisms from regulation (as GM) or established fast-tack procedures, with many of these being major producers and exporters of agricultural commodities, whereas the non-adopters (of which Japan is an example) rely on imports of agricultural commodities from the adopters. The authors examine the changing policy dynamics in these jurisdictions, which include pressure to improve agricultural sustainability.

Collectively, the articles in this Research Topic demonstrate the potential for genome editing to have a prominent role in improving the sustainability of agriculture, with broader implications for the global challenges of food security and nutrition. A strong theme throughout these articles is the need for an enabling regulatory environment if this potential is to be realised. Keiper and Atanassova examine the evolving global regulatory landscape and its implications for commercialising improved crop varieties developed using genome editing. The regulatory challenges that currently exist for globally traded GM crop commodities, and the resulting restrictions on technology adoption and innovation, are highlighted to contend that genome editing provides an opportunity to revise regulatory policies to support a more proportionate and enabling approach.

